# Breast surgery: a narrative review

**DOI:** 10.5694/mja2.51678

**Published:** 2022-08-21

**Authors:** Christobel M Saunders

**Affiliations:** ^1^ The University of Melbourne Melbourne VIC

**Keywords:** Breast surgery, Cancer, Breast neoplasms

## Abstract

- Breast cancer is the commonest human cancer globally and one in seven Australian women will develop it in their lifetime.
- Surgery is the mainstay of management both for women who are at high risk of breast cancer and for those who have been diagnosed.
- Increased understanding of how to predict who is most at risk of breast cancer is leading to the possibility of risk‐based screening, allowing better and more targeted early detection for women at high risk, and contrast imaging techniques are proving more accurate in diagnosing and staging cancer.
- The evolution of surgical practice includes the widespread use of oncoplastic surgery, allowing better cosmetic and oncological outcomes; reconstructive surgical advances, using free flap techniques; and sequencing of systemic and local therapies to better tailor treatments to the patient’s cancer and improve outcomes.
- Recognition of side effects of breast cancer treatment have led to improvement in the management of conditions such as chronic pain and lymphoedema, as well as addressing the psychosocial, body image and sexual complications caused by the cancer and its treatment.

Breast cancer is the commonest human malignancy and Australia and New Zealand have the world’s highest age‐standardised incidence rate, translating to one in seven Australian women diagnosed with breast cancer in their lifetime.^1^ Each year, over 20 000 Australians develop breast cancer (which includes nearly 200 men),^2^ about a third of whom are diagnosed through population or private screening. However, many patients present to general practitioners with a breast symptom that is not cancer, often requiring investigation and referral to a specialist surgeon. Survival from breast cancer has increased by more than 20% over the past two decades, and 93% of people diagnosed today will be alive and disease‐free 5 years after diagnosis.^2^ This means that there are 200 000 Australian women (and about 200 men^3^) living with a prior diagnosis of breast cancer, many of whom continue to have significant morbidity from their disease and its treatment. Thus, the absolute disease burden of breast disease is significant in the Australian community.

Surgical management, within the context of multidisciplinary care, is the mainstay of breast cancer therapy, and perhaps for the majority of patients it is likely curative in itself, although most will have adjuvant radiotherapy and drug treatments (Box 1).^4^ Unfortunately, to date, we have no precise way to predict who could avoid further non‐surgical treatment. Australian research has been in the forefront of the move to develop more targeted therapies, de‐escalating unnecessary and potentially toxic treatments, such as the EXPERT trial, which is identifying who can avoid radiotherapy,^5^ or the TARGIT trial of a single dose of intraoperative radiotherapy.^6^ Surgery is not practised in isolation but relies on a large team of health professionals, with the patient at the centre, and vital roles played not just by the cancer clinicians but notably by breast care nurses, allied health professionals and general practitioners.

Box 1 **Pathways from diagnosis through treatment for breast cancer**

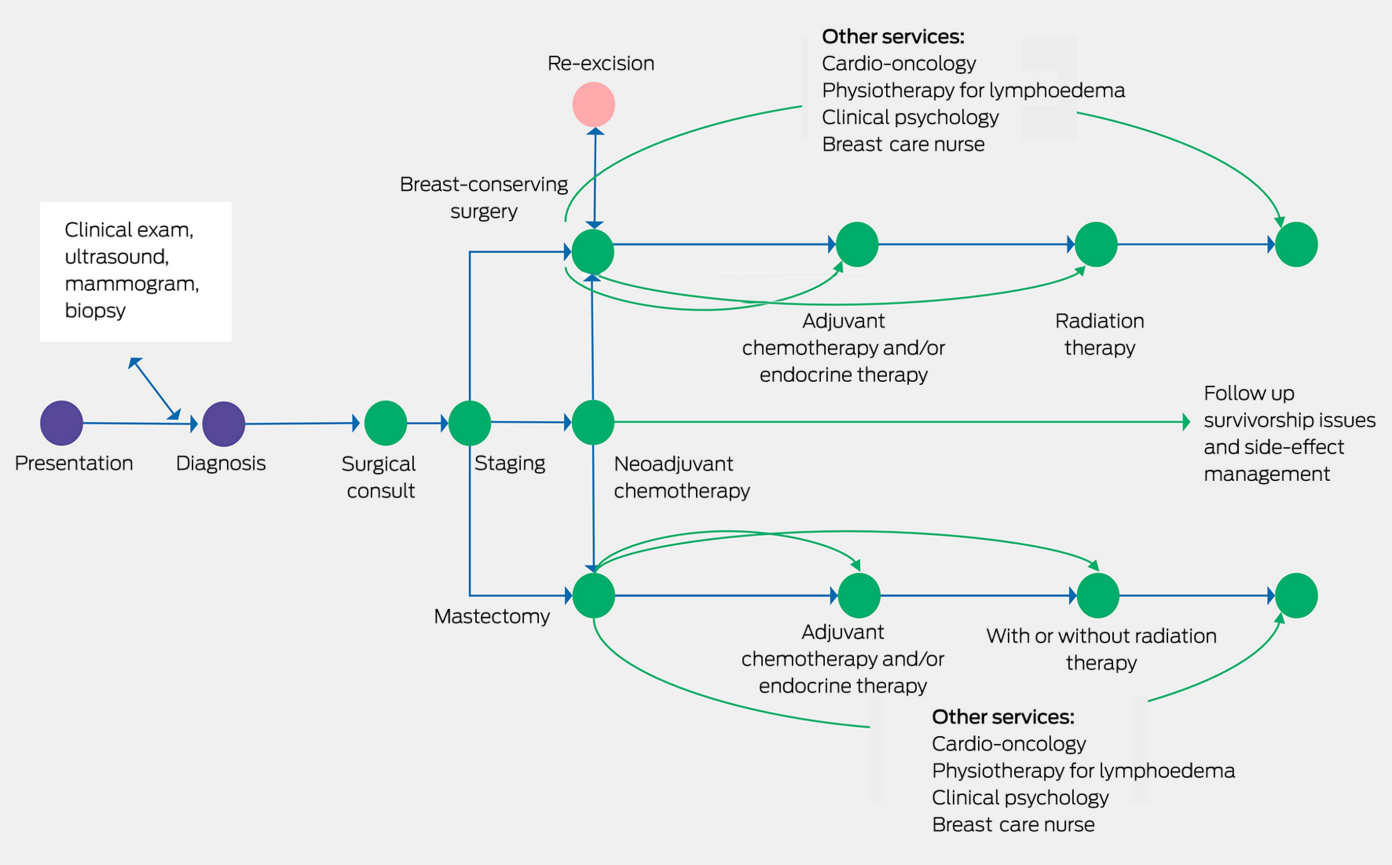

This narrative review highlights some recent advances in the prevention, diagnosis, and loco‐regional management of breast cancer, and shows why it is imperative to offer the most efficient and effective treatments and to continue to research better ways of breast cancer treatment and prevention. This article is based on evidence collected to inform the most recent Cancer Australia guidance for the management of early breast cancer,^7^ which used a meta‐guideline approach with an expert review panel distilling evidence from international guidelines and systematic reviews from 2007 onwards.

## Prevention and screening

Most breast cancers have no obvious known cause. Even though we can identify population‐level risk factors, such as exposure to endogenous and exogenous female hormones, it is only in the minority of breast cancers, associated with high penetrance familial mutations such as *BRCA1* and *BRCA2*, that prediction of risk is somewhat accurate.^8^ For many of these individuals, surgical prevention (bilateral mastectomy) is the only proven technique to reduce the risk of developing or dying from breast cancer.^9^ However, recent understanding of the oncogenesis of breast cancer (BRCA)‐related cancers has suggested that modifying the receptor activator of nuclear factor‐κB (RANK) ligand pathway with the drug denosumab may prevent cancer formation,^10^ and a clinical trial (BRCA‐P)^11^ is underway to test this hypothesis. For individuals with a family history but no proven genetic mutation or who have other risk factors (eg, atypical but non‐malignant breast lesions), oestrogen receptor modulators (eg, tamoxifen) and the aromatase inhibitor drugs can more than halve the risk of developing oestrogen receptor‐positive breast cancer,^12^ although a decrease in all‐cause mortality is still elusive.

Newer risk factors, notably mammographic density and single nucleotide polymorphism combinations, used alongside more traditional risk factors may enable us to describe a phenotype in which a person has considerable increased risk.^13^, ^14^ Lifestyle modification, including maintenance of healthy weight, exercise and minimising alcohol intake, will decrease risk, and appropriate surveillance can detect cancers earlier.^15^

Risk stratification holds the promise of targeted surveillance — so‐called risk‐based screening. Current population screening by a mammogram every 2 years targets all women aged 50–74 years, with age as the only risk taken into account.^16^ This strategy has resulted in a sharp increase in detection of early stage cancers, both *in situ* disease and small, favourable outlook cancer amenable to breast‐conserving surgery. Furthermore, the strategy delivers a relative overall survival advantage of over 20% for women who participate in screening.^16^ However, the benefit of screening in both mortality reduction and earlier detection is markedly different for different risk groups. There is undoubtedly overdiagnosis and false positive recalls,^17^ which result in unnecessary investigation and treatment of some women and in less than optimal screening of those at high risk.

Work is underway in Australia to define how best to stratify women according to risk, and how best to develop pathways, led by both general practice and BreastScreen, to adapt screening protocols to risk.^18^ Two key questions are how to measure risk and what supplemental screening should be offered to women with risk features such as high mammographic density, which is an increased risk factor and makes cancer detection more difficult.

Another concern being addressed in screening is equity. Currently, about 54% of the eligible population participate in BreastScreen, although other women will undergo surveillance outside the BreastScreen program.^16^ But we know that fewer Indigenous and non‐English‐speaking women and fewer women living in remote locations participate in screening, and all experience worse cancer outcomes.^16^ The future of better prevention and screening will be risk stratification and appropriate management based on this. However, until further research clarifies what this should look like, we continue to recommend healthy lifestyle and BreastScreen programs for most Australian women.

## Diagnosing breast disease

Breast symptoms are common and most will turn out to be benign. An important simple formula is the “triple test”,^19^ whereby any woman with a persisting and worrying breast symptom requires a clinical examination, appropriate imaging, and tissue biopsy to all be concordant in order to exclude a potential malignancy.

The mainstay of imaging has been ultrasound with or without mammography, increasingly with tomosynthesis. Newer contrast‐enhanced techniques are proving more sensitive and more accurate in specialist centres.^20^ Mammography using iodine contrast is fast becoming an important tool that can better delineate known lesions but also pick up otherwise occult areas of cancer, especially in dense breast tissue.^20^ Magnetic resonance imaging (MRI) with gadolinium contrast has been shown to be better for screening very high risk younger women, but is also useful in problem solving for some women diagnosed with breast cancer. An abbreviated MRI may offer similar information but prove quicker, cheaper and, thus, more widely applicable.^21^ Molecular breast imaging uses technetium‐99m sestamibi, and is more of an adjunct once an abnormality is found on conventional imaging, especially in dense breasts.^22^ As it exposes the whole body to radiation, it is not an ideal screening tool.

Positron emission tomography (PET) is now widely used to stage for distant metastases,^23^ and can be adapted to the breast (positron emission mammography) to detect small areas of functional abnormality, or even used with oestrogen‐linked tracers to confirm metastatic oestrogen receptor‐positive cancers.^24^ However, radiation and expense precludes this as a good screening test.

Optical imaging is emerging not so much as a diagnostic tool but as one to help guide surgery. One promising Australian technology has been shown to be able to detect microscopic residual tumour at the margins of surgical excision^25^ and could thus prove very useful in ensuring all tumour is removed at one operation. Technologies using fluorescence or other optical modalities are also under investigation.

Circulating tumour markers, most commonly circulating tumour DNA (ctDNA) or circulating tumour cells, have been proposed as an adjunct in screening,^26^ but as yet this is a long way off reality. However, these markers are beginning to demonstrate their utility in early detection of recurrent cancer before other clinical or imaging tests are positive.^26^ Whether detecting recurrence this early changes disease outcome, by allowing earlier or better targeted treatment, is yet to be determined.^27^ Any new breast symptoms should be subjected to the triple test, and if cancer is diagnosed, contrast imaging should be considered, especially in younger women and those with increased breast density.

## Treating breast cancer: evolution of surgical management

Surgery has been the primary management modality for breast cancer for centuries and indeed the radical (or Halsted) mastectomy, devised at the end of the 19th century,^28^ was used almost exclusively until the 1900s. Randomised trials confirmed that, for many women with breast cancer, breast‐conserving treatment (“lumpectomy”, usually with whole breast radiation) is at least as good if not superior to mastectomy in terms of survival, with excellent local control.^29^ In Australia, just under 70% of women undergo breast conservation.^30^ Mastectomy is largely reserved for those with large volume or multifocal disease, if radiation is contraindicated, or for patient preference.^30^ If mastectomy is undertaken, breast reconstruction can often be offered, which is either done by the breast surgeon or in combination with a plastic reconstructive surgeon. Reconstruction after mastectomy, especially if done at the same procedure, improves psychological wellbeing.^31^ Reconstruction techniques include the use of implants — often with an initial tissue expander first and covered by a synthetic or biological matrix material acting as an “internal bra” — or autologous flaps which offer excellent cosmesis. The technique used needs to be tailored to the individual, her body shape and size, comorbid conditions, and expectations. A reconstructed breast is only ever a facsimile of the woman’s natural breast, although using native tissue and, if possible, preserving the nipple gives a more realistic outcome with better patient satisfaction.^32^

Most Australian breast surgeons now practise oncoplastic techniques, which essentially aim to remove the tumour while giving optimal cosmetic outcomes. This means careful scar placement, volume redistribution or bringing in local flaps to replace the lost volume. It may mean that the contralateral breast also needs symmetry surgery, for example, if a reduction procedure is done on the cancer side to remove a large tumour. Furthermore, with these techniques, much larger cancers can now be removed with good aesthetic and functional results.^33^ Increasingly, we are assessing the outcomes of surgery on a more routine and systematic basis using patient‐reported outcome measures,^32^ which it is hoped may add a quality measure to surgical practice.

A key to deciding on the ideal operative management is accurate and sensitive imaging. As discussed above, some of the newer modalities, such as contrast mammography and MRI, have proven useful to locally stage breast cancer and help establish what type of surgery (and when to perform it) is the best option, although studies are still underway to optimise their use.^34^

A third of breast cancers are diagnosed through screening programs,^16^ and often these are small and impalpable tumours that need localisation techniques in order for the tumour to be effectively removed at surgery.^16^ This was traditionally done by placing one or more hookwires into the lesion under imaging control on the day of the surgery. A cluster of new techniques have more recently come on to the market, including magnetic and radar markers^35^, ^36^ and tiny radioactive seeds, all of which can be placed days or weeks in advance, thus decoupling the localisation from the surgery and making the workflow much smoother. Not all of these, however, are funded, all have advantages and disadvantages, but only radioactive iodine seeds have been shown to be more accurate than hookwires in a randomised trial, thus decreasing re‐excision rates.^36^

About 30 years ago, it was postulated that delivering chemotherapy upfront before surgery may have a number of advantages over the usual policy of surgery first. This neoadjuvant approach is now offered to 10–20% of patients diagnosed with breast cancer.^37^ The main aims are, first, for patients with large or inoperable tumours, such as inflammatory cancer, to become operable by mastectomy.^37^ Second, patients with large tumours requiring mastectomy can be downstaged to allow breast‐conserving surgery, and in some cases a good response in axillary nodes can then allow less extensive targeted axillary dissection. Third, the tumour response to drugs *in situ* can allow tailoring of systemic treatment. Neoadjuvant chemotherapy is particularly effective for “triple negative” (ie, not expressing oestrogen, progesterone, or human epidermal growth factor receptor 2 [HER2] receptors) and HER2‐positive cancers (overexpressing the growth factor HER2),^38^ measured both on the initial core biopsy and subsequent surgical resection. Up to 50% of these patients will achieve a complete pathological response from drug therapy which translates into a very good survival risk.^38^ Moreover, for patients who have residual disease after chemotherapy when the tumour bed is resected, other drug options may be available after surgery to improve relapse rates.^39^, ^40^ Another important role of neoadjuvant drug therapy is to act as an *in vivo* “test bed” in studies that gauge the tumour response to new therapies.^38^ Radiotherapy is also sometimes used before surgery, particularly in women in whom mastectomy and immediate autologous reconstruction is planned,^41^ to facilitate a single stage surgical procedure.

Involvement of regional axillary lymph nodes remains one of the most powerful prognostic indicators in early breast cancer,^7^ thus staging of the axilla by removing some lymph nodes has long been standard surgical practice. Current guidelines mandate that for people who do not have obvious clinical or radiological evidence of axillary lymph node involvement before surgery, sentinel node (SLN) biopsy rather than axillary clearance should be offered.^42^ SLN biopsy uses a radioactive tracer and/or blue dye to identify the lowest nodes draining the breast and facilitates the removal of just this one or a few nodes, thus minimising the toxicity of axillary clearance, including lymphoedema and arm function deficits.^7^ However, recent trial evidence suggests that, even if the SLN does have a small volume of cancer within it, no further axillary treatment is needed.^43^ Management of the axilla continues to evolve, essentially progressing to less invasive treatments, as we continue to better understand the biology of the primary tumour to guide treatment decisions rather than needing nodal staging information. SLN biopsy is standard of care for patients with no known nodal disease, and even with the presence of small volume metastases in axillary nodes, further surgery is rarely needed.

## Managing some side effects of cancer surgery

Breast and axillary surgery is generally well tolerated,^44^ with most patients admitted to hospital as a day case or overnight. Nevertheless, the longer term consequences, including the most common ones of pain and lymphoedema, can cause many years of toxicity.^45^ Psychosocial fallout from breast surgery, including effects on body image and sexuality,^45^ are a very important consideration. The long term physical effects of surgery include chronic pain in up to 60% of women.^46^ This effect is being addressed by a number of techniques, including perioperative intravenous lidocaine, in the Australian multisite LOLIPOP trial (ClinicalTrials.gov, NCT05072314).

Lymphoedema is a much feared side effect of axillary clearance and/or radiotherapy and occurs in up to 25% of women who have these treatments.^47^ Good education and prevention are now commonplace, with most women seeing a physiotherapist or lymphoedema specialist both before surgery and if symptoms develop.^7^ For patients with intractable lymphoedema, some Australian plastic surgeons are pioneering lymph node transfer and similar techniques with great promise.^48^ Early referral to a lymphoedema specialist for exercise advice and prevention strategies, including avoidance of arm infections, is important to optimise recovery after breast cancer surgery and minimise side effects such as lymphoedema.

## How new insights into breast cancer biology are affecting surgical management

Understanding the biology of tumours is making inroads in clinical cancer practice. It can facilitate targeted treatments and can change either the type of surgery or the sequence in which we undertake surgery or lead to such good cancer outcomes that less extensive surgery is needed. An early example of drugs that exploit these insights include the first cancer monoclonal antibody, trastuzumab, which, along with newer HER2‐targeted drugs, is highly effective for the 12% of breast cancers that overexpress the HER2 receptor.^49^ Newer agents include drugs targeting the cyclin‐dependent kinase (CDK) 4/6 pathway^50^ and poly (ADP‐ribose) polymerase inhibitors (PARPi)^51^ in BRCA‐related cancers. Recent studies showing the survival benefits of PARPi are presenting powerful arguments for offering germline genetic testing to more newly diagnosed patients with breast cancer.^51^ This testing may be offered even without a family history of cancer, as finding a mutation such as *BRCA1* or *BRCA2* will trigger both tailored surgery (eg, bilateral mastectomy instead of breast‐conserving surgery, and risk‐reducing oophorectomy) and targeted drugs.

Understanding immune escape mechanisms and the tumour micro‐environment is bringing immunotherapy closer for some women with HER2‐positive and weakly oestrogen receptor‐positive and, notably, triple negative breast cancers, where the cancer cells express programmed death‐ligand 1 (PD‐L1).^52^ This molecule attaches to programmed cell death protein 1 (PD‐1) receptors on T cells, rendering the immune system less able to attack the tumour, and this may be reversed by immunotherapy.^52^ It is hoped that these drugs may allow less extensive or even no surgery into the future.

Precision drugs such as those described above need to be carefully orchestrated with loco‐regional surgery and radiotherapy; for example, deciding if these agents are best used after the tumour is removed at primary surgery to allow proper assessment of pathology, or giving drugs upfront when the extent, timing and sequencing of surgery and radiotherapy are still not clear. Coupled to this is the need to better understand the treatment toxicities and interactions of these newer therapies and how we can manage them to optimise patient outcomes.

## Surgery in metastatic disease

Although most patients who develop distant metastatic disease will not ultimately be cured,^53^ many will still have very long survival trajectories,^54^ and the aims of treatment are both to prolong survival and to optimise the quality of life.^55^ Surgical resection is sometimes offered to prevent serious complications,^55^ such as stabilising a spine with vertebral metastases or removing a fungating breast tumour, but can also decrease tumour load and allow more effective systemic treatment such as with small volume brain or liver metastases. Research is still needed to determine if surgical intervention in metastatic disease improves survival.

## Future possibilities

Despite impressive new systemic cancer treatments, surgery is likely to remain the mainstay for most people diagnosed with breast cancer into the future. However, it is likely to evolve, so de‐escalation of treatments means that perhaps we can look to a future of minimal breast‐conserving surgery only for some patients, accurately predicting no need for more treatment. Trials of percutaneous outpatient surgery using, for example, cryoablation, radiofrequency, microwave or laser ablation, or vacuum‐assisted excision, are also promising.^56^ For some tumours with specific mutations or immune environments, drug treatment alone may offer cure without the need for surgery. In the medium term, surgery will remain the primary treatment for nearly all patients diagnosed with breast cancer, performed by highly subspecialised surgeons who have undertaken extensive training both in technical aspects of breast and reconstructive surgery but also in multidisciplinary oncology care (www.breastsurganz.org). Moreover, the strong and respectful links between surgical practice and patients, both individually and via remarkable patient advocacy organisations (www.bcna.org.au), will continue to drive patient‐centred, value‐based care and research, which will improve survival rates in the coming decades.

## Competing interests

Christobel Saunders receives consultancy fees from Merck Sharp and Dome, and is the Chief Medical Officer of OncoRes, from which she perceives no salary or benefits, and the shares are lodged with the University of Western Australia.

## Provenance

Commissioned; externally peer reviewed.

## Open access

Open access publishing facilitated by The University of Melbourne, as part of the Wiley ‐ The University of Melbourne agreement via the Council of Australian University Librarians.
